# First evidence for fin whale migration into the Pacific from Antarctic feeding grounds at Elephant Island

**DOI:** 10.1098/rsos.220721

**Published:** 2022-09-21

**Authors:** H. Herr, L. Hickmott, S. Viquerat, S. Panigada

**Affiliations:** ^1^ Institute of Marine Ecosystem and Fishery Science, Center for Earth System Research and Sustainability, University of Hamburg, Große Elbstraße 133, Hamburg 22767, Germany; ^2^ Alfred Wegener Institute Helmholtz Centre for Polar and Marine Research, Am Handelshafen 12, Bremerhaven 27570, Germany; ^3^ Sea Mammal Research Unit, Scottish Oceans Institute, University of St Andrews, Fife KY16 8LB, UK; ^4^ Open Ocean Consulting, 3b Oaklands Road, Petersfield, Hampshire GU32 2EY, UK; ^5^ Tethys Research Institute, Viale G.B. Gadio 2, Milan 20121, Italy

**Keywords:** satellite telemetry, Southern Ocean, *Balaenoptera physalus*, population connectivity, migratory routes

## Abstract

This study presents the first long-distance tracks of fin whales (*Balaenoptera physalus*) equipped with satellite transmitters off the Antarctic Peninsula. Southern Hemisphere fin whales were severely depleted by twentieth century industrial whaling, yet recently, they have returned to historical feeding grounds off the northern Antarctic Peninsula, forming large aggregations in austral summers. To date, our knowledge only extended to summer behaviour, while information regarding migration routes and the location of breeding and wintering grounds are lacking. During the austral autumn of 2021, we deployed nsatellite transmitters on four fin whales at Elephant Island. Two transmitters stopped working while the animals were still at the feeding grounds, while two continued to transmit during the transition from feeding activity to migration. Both migrating animals left the feeding ground on 15 April 2021, travelling northward into the Pacific and up along the Chilean coast. The most northerly position received before all tags stopped transmitting on 1 May 2021 was at 48°S. These tracks provide initial evidence of seasonal migratory routes and a first indication toward possible locations of winter destinations. This information, even if preliminary, is critical for investigations of population connectivity, population structure and the identification of breeding grounds of Southern Hemisphere fin whales.

## 1. Introduction

During the twentieth century, Southern Hemisphere fin whales (*Balaenoptera physalus*) were targeted heavily by industrial whaling, with approximately 723 000 individuals killed [1] prior to the end of their commercial exploitation in the mid-1970s [2]. Whaling operations largely focused on (sub)Antarctic feeding grounds around South Georgia and the Antarctic Peninsula, where fin whales aggregated in large numbers during austral summers [3,4]. Whalers knew that fin whales moved out of Antarctic waters at the end of the feeding season, and that some reached subtropical to tropical waters [5]. However, information on how far the majority travelled towards the equator, and whether they dispersed or concentrated in definable regions for breeding in winter was not known of [5]. At the end of the commercial whaling period [2], few fin whales were left in the Southern Hemisphere [6]. It is thought that the population had been reduced to 1–2% of its pre-exploitation size of formerly approximately 325 000 animals [1,6,7]. Only a few sightings of fin whales were reported from historical feeding grounds for decades and only in the 2000s did observations of larger numbers of fin whales start being reported again from the northern Antarctic Peninsula [8,9]. Hotspots of fin whale occurrence were identified near Elephant Island, based on data collected during repeated krill surveys [10], and dedicated cetacean surveys confirmed high fin whale densities at historical feeding grounds [11–13]. Furthermore, large feeding aggregations up to 150 animals were observed in these locations [11,13–15], suggesting at least some level of population recovery [13]. While fin whales have apparently returned to some of their ancestral feeding grounds, their breeding or wintering grounds have not yet been identified.

**Figure 1.**
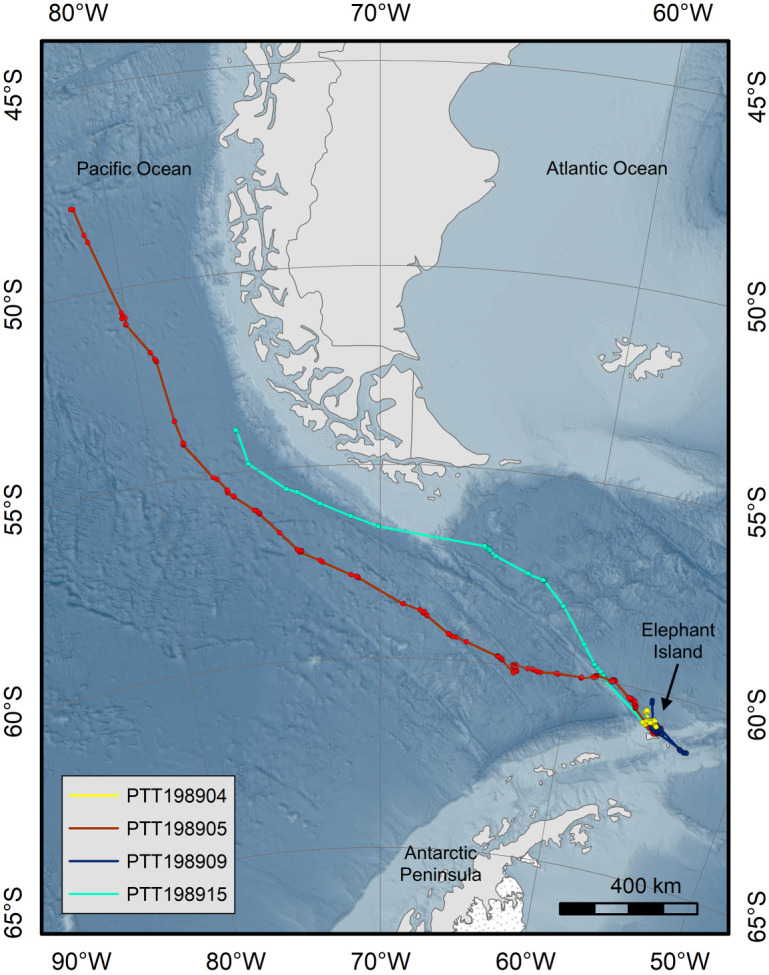
Display of tracks of four fin whales tagged off Elephant Island (Western Antarctic Peninsula). Two fin whales migrated from the tagging site to the west coast of South America, crossing the Drake Passage into the Pacific Ocean. A close-up view of all tracks at the tagging site is provided in the electronic supplementary material, figure S2.

Fin whales of the Southern Hemisphere are thought to belong to one subspecies, *B. physalus quoyi* [16], but their finer-scale population structure is still poorly understood [16,17]. Therefore, whether fin whales feeding at Elephant Island belong to one or several breeding populations is still unknown. In the case of humpback whales (*Megaptera novaeangliae*), genetically segregated breeding stocks from the Pacific and from the Atlantic mix at Southern Ocean feeding grounds [18,19]. Fin whales occur in both the South Pacific and the South Atlantic Oceans [5,20,21], therefore both basins are candidate areas for migratory destinations of fin whales gathering off the northern Antarctic Peninsula during summer (figure 1).

In our study, we deployed satellite transmitters on fin whales at Elephant Island for the first time, in order to track their movements at the end of the feeding season and to shed first light on possible migratory destinations.

## 2. Methods

Satellite transmitters were deployed on fin whales at the northwestern coast of Elephant Island (61°S, 55°W), Antarctica, in March and April 2021. Tagging operations were accomplished during a five-week expedition aboard the 23 m sailing vessel ‘Australis’, which had the additional purpose of filming fin whale feeding aggregations. Aggregations in this context are defined as extremely large groups (greater than 15 animals) with individuals not further apart from each other than 5 body lengths (see also [13]). Locations of aggregations (position of ship when next to the aggregation) were noted and aggregation sizes were assessed visually, with film and drone footage used to confirm the visual assessments [13]. Tagging was conducted upon an encounter of a fin whale aggregation during favourable weather conditions, from a 4.7 m rigid-keeled inflatable boat. Individuals to be tagged were chosen opportunistically, adhering to best practice tagging guidelines by Andrews *et al.* [22]. Low-Impact Minimally Percutaneous External Electronic (LIMPET) tags carrying either SPLASH10-333 or SPLASH10-F-333 GPS Fastloc® transmitters from Wildlife Computers were deployed using a crossbow (Excalibur APEX XLT, 150 lb draw weight). The small (70 g) LIMPET tags were each attached by a pair of sterilized barbed titanium darts on the dorsal fin, at distances of 5–15 m [22]. Tags were programmed to transmit over 1–5 h intervals each day, up to 14 h of total transmission time and a maximum of 300 transmissions per day. Tag locations were obtained from the ARGOS satellite system (CLS, France) which rates location quality B, A, 0, 1, 2 or 3 in increasing order of position accuracy. Estimated error for class 3 is predicted at less than 250 m by the ARGOS service, for 2 = 250–500 m, for 1 = 500–1500 m, for 0 = greater than 1500 m [23]. For A and B no accuracy estimation is available. Since the focus of this analysis was the long-range movement of fin whales after leaving their feeding grounds, fine-scale movements and small-scale position accuracy were of lower priority, and we used classes 3, 2, 1 and 0 for the visualization and analysis of tracks.

## 3. Results

During the expedition, 11 feeding aggregations were encountered, ranging in size from 25 to approximately 300 animals (electronic supplementary material, figure S1 and table S1). During these aggregation events, seven tags were deployed on adult fin whales between 29 March and 10 April 2021 (table 1). Unfortunately, three transmitters failed to send any messages, despite one of these tags being resighted, still well attached as deployed, 5 days after deployment. Transmission duration of the four active tags ranged from 4 to 29 days (table 1 and figure 1). The mean number of locations received per day was 23.2 ± 8.90 across all location quality classes, and 8.6 ± 5.22 after exclusion of classes A and B.

**Table 1.** Satellite transmitter deployment information.

deployment	tag-ID	tag type	last Location	tracking duration	locations received	locations received classes 0–3	comments
Date, Time (UTC) Position (Lat, Lon)			Date, Time (UTC) Position (Lat, Lon)		total (daily mean ± s.e.)	total (daily mean ± s.e.)	
28 Mar 21, 20:03	PTT198904	Standard Mk10	31 Mar 2021, 01:13:30	4 days	54 (13.5 ± 5.41)	40 (10 ± 3.81)	
−60.89374, −55.41108			−60.498, −55.697				
03 Apr 21, 17:27	PTT198905	Standard Mk10	01 May 2021, 10:15:22	29 days	715 (24.7 ± 9.39)	344 (11.9 ± 4.18)	resighted and photographed on 4-Apr-21
−61.00403, −55.09396			−47.869, −81.322				
10 Apr 21, 17:07	PTT198909	Standard Mk10	22 Apr 2021, 03:38:58	13 days	257 (19.8 ± 5.98)	32 (2.46 ± 1.87)	
−61.04523, −54.95442			−60.002, −55.770				
10 Apr 21, 20:19	PTT198915	Fast-Loc Mk10	25 Apr 2021, 13:19:23	16 days	411 (25.7 ± 8.32)	118 (7.3 ± 3.94)	
−61.06432, −54.89974			−53.880, −76.233				

**Figure 2.**
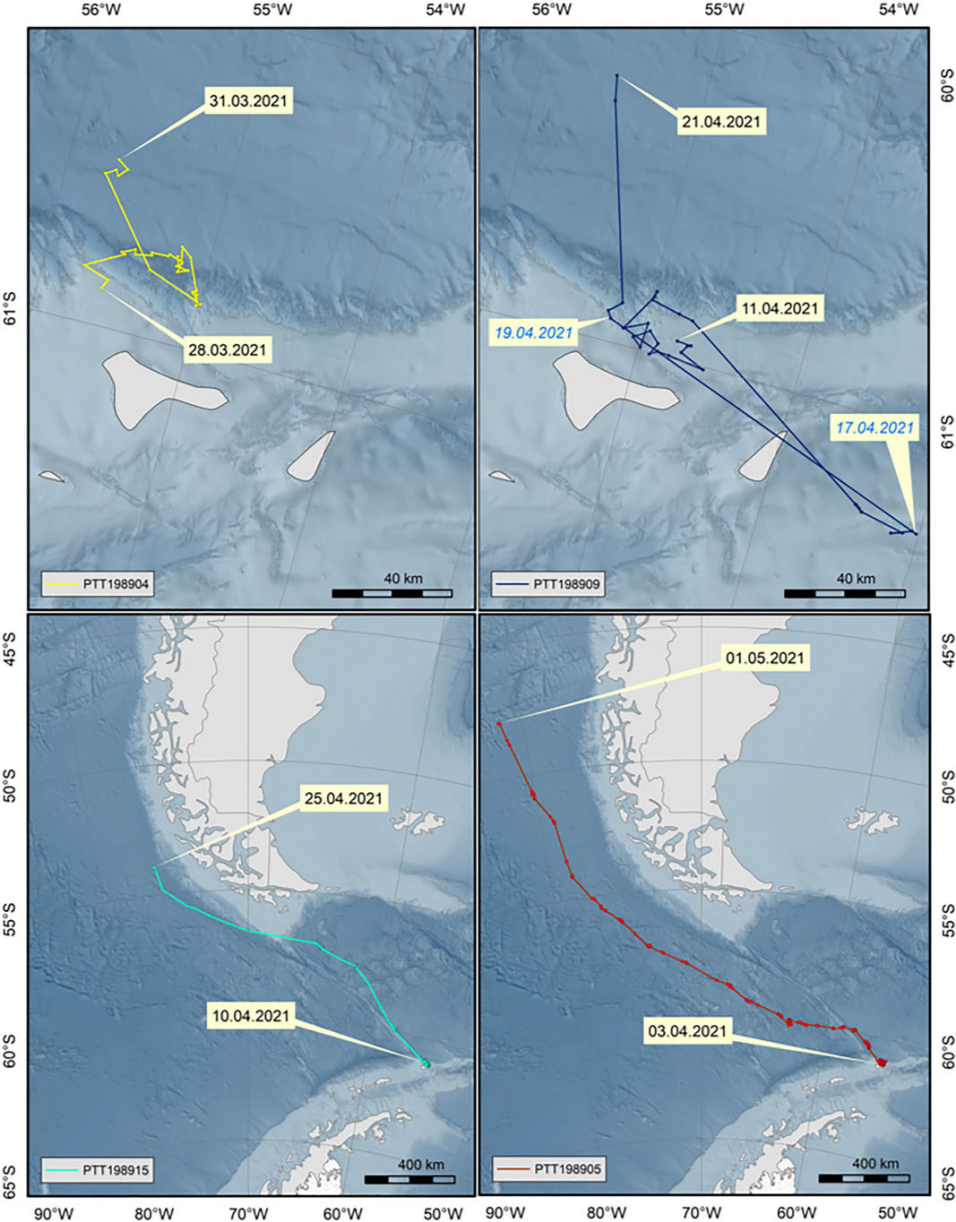
Tracks of each whale tagged at Elephant Island, showing the dates of first and last transmission (black dates), and in the upper right panel also the dates marking the fin whale's change of course to return back to the tagging site and starting migration (blue dates). The upper two panels are close-up sections of Elephant Island and the neigbouring smaller Clarence Island.

After tagging, all four animals remained on the feeding ground until 15th April (electronic supplementary material, figure S2). One instrument (PTT198904) stopped transmitting after 4 days while the animal was still resident at the feeding ground (figure 2; electronic supplementary material, figure S2). The remaining three animals left the area on the same day (15 April 2021): two (PTT198905 and PTT198915) to the northwest and the third to the southeast (PTT198909) (figure 2). Having travelled to the southeast, PTT198909 then returned to the tagging site after 3 days (18 April 2021). It then began following a similar course to the northwest, akin to the routes of the other two whales (figure 2). However, shortly after picking up this course, the tag stopped transmitting on 22 April 2021 (a total of 13 transmission days). PTT198905 and PTT198915 continued transmitting, following a relatively straight course north–northwest (figures 1 and 2). PTT198915 crossed the Drake Passage in 5 days, reaching the tip of South America (S51.17 W67.03) on 20 April 2021. From there it continued into the Pacific and moved up the Chilean coast along the shelf edge (figure 2). Transmissions stopped after a total of 15 days at S53.89, W76.23. PTT198905 crossed the Drake Passage in 11 days and stayed further from the South American coast than PTT198915. Travelling parallel to the course of PTT198915 into the Pacific, this animal was tracked further north to S47.87 (figure 2), where the tag stopped transmitting after 29 days. Since leaving Elephant Island to the end of transmissions, PTT198905 covered a distance of 2300 km in 16 days, an average 144 km d^−1^.

## 4. Discussion

Our study provides the first evidence for a migration of fin whales from the feeding grounds at Elephant Island into the Pacific Ocean and a first indication of a migration route. Deployment of satellite transmitters at the end of the feeding season allowed us to track the beginning of their northward migration. However, transmissions stopped while the animals were still in transit, therefore their final migratory destination can only be hypothesized. An equatorial hiatus, a gap in the global distribution of fin whales observed between approximately 20°N and 20°S [21], suggests a migratory destination in the South Pacific. A high prevalence of cookiecutter shark (*Isistius* spp.) bite lesions observed in fin whales feeding at Elephant Island [24]) suggests migration to latitudes north of 41°S, the most southerly distribution of *Isistius brasiliensis* [25]. In the eastern Pacific cookiecutter sharks are known to occur around the Galapagos, Easter and Guadalupe Islands [26]. A possible breeding ground for fin whales in the eastern subtropical Pacific (21°27′S, 97°34′W, 2810 km off the Chilean coast) has been suggested based on an opportunistic observation of a large dispersed group of fin whales at the end of May in 2010 [20]; however, to date, this was a singular observation and no breeding behaviour has been described from this region, or elsewhere in the South Pacific. Information on the distribution and movements of fin whales in the southeast Pacific Ocean is primarily available from whaling operations conducted in the twentieth century [1,27]. Along the Chilean coast, fin whales were caught both in coastal and oceanic waters [27]. Several fin whales marked with discovery tags off the Chilean coast during whaling operations were later caught in Antarctic waters [27]. Therefore, Chilean waters were regarded as a migratory corridor between lower latitude breeding grounds and Antarctic feeding grounds of fin whales [3,5,27]. However, in the post-whaling era, fin whales have been confirmed to occur all along the Chilean coast, predominantly in offshore waters, but also in coastal areas [28–31]. Acoustic studies have suggested population connectivity between fin whales recorded off the coast of Chile and the Antarctic Peninsula [15] based on spectral similarities in fin whale calls [15,32]. However, more recent fin whale studies have proposed the existence of spring and summer foraging areas of fin whales in coastal habitats off Chile, suggesting that not all fin whales in Chilean waters migrate to higher latitudes to feed [30,33]. Tracking of six fin whales tagged off the Chilean coast in November/December 2015 revealed only one fin whale with a clear southbound migratory behaviour, with the others remaining at middle-latitudes for the whole time the tags transmitted (up to 77 days), suggesting at least temporal residency [29]. Therefore, as a result of the current literature and knowledge base, it is difficult to deduce the migratory destination of the individuals feeding at Elephant Island based on observations of fin whale occurrence in the South Pacific; particularly, because fin whales may not concentrate in specific breeding grounds during winter, but rather disperse throughout a wider area. Whalers came to this conclusion based on a lack of observations of fin whale concentrations anywhere else than on the feeding grounds [5]. More deployments are needed to follow the fin whales’ migration from Elephant Island in order to reveal how far they travel, and if they reach a common destination or disperse to differing stock locations. By tracking additional fin whales in the future, it may be possible to establish if the animals feeding at Elephant Island all migrate into the Pacific, or whether fin whales from both the Atlantic and the Pacific mix at Antarctic feeding grounds. In the twentieth century, fin whales from the South Atlantic were assumed to migrate to Antarctic waters [5]. Large numbers of fin whales were caught in the South Atlantic, particularly around South Georgia [4,5]. Today, fin whales are again observed in South Georgian waters [34] and could potentially also migrate to the Antarctic Peninsula, but their movements have not yet been investigated. However, long-term acoustic recordings of fin whale song off Elephant Island show very little change over time, supporting the hypothesis of a single, stable population at the Western Antarctic Peninsula that has been consistently using the area and that is rarely exposed to foreign individuals [35].

Genetic analysis of biopsy samples collected from fin whales may also help to gain insights into population structure and the likelihood of populations mixing on the feeding ground. Recent phylogenetic analyses, however, have shown an absence of strong genetic structure in fin whales of the Southern Hemisphere [16]. In the Northern Hemisphere, genetic differentiation between the Atlantic and the Pacific is strong [17,36]. A pattern that has been observed also for humpback whales [36] and which is likely related to continents separating ocean basins and preventing inter-oceanic dispersal [16]. In the Southern Hemisphere, low genetic differentiation and high gene flow among regions have been reported for humpback whales despite the existence of discrete breeding stocks [37]. Therefore, information on Southern Hemisphere fin whale breeding populations using genetic methods may be limited.

The results of our study provide preliminary insights on the timing of the northward migration of fin whales from Antarctic feeding grounds, indicating that animals start leaving during mid-April. These results are supported by historical whaling data and visual records. In the twentieth century, whaling for fin whales was carried out around the South Shetlands (including Elephant Island) from December (rarely November) to April, with highest catch numbers from January to April, peaking in March [4], likely representing the abundance of fin whales in the area [38]. Visual records of fin whales confirm presence at Elephant Island from December to April [8,11,13,39,40]. However, visual records, as well as whaling activities in the Southern Ocean, are likely biased towards the summer months, when most expeditions to the Antarctic are carried out [4,8,41]. Acoustic recordings have shown peak levels of fin whale calling activity around Elephant Island between April and June, with a steep decrease from July/August onwards [15,42,43], suggesting that either fin whales leave the area later than indicated by visual records, or that calling activity may be associated with migratory behaviour. Our tracks show at least three fin whales to leave the feeding ground at Elephant Island in mid-April. Based on the small sample size, conclusions are drawn with caution. The fact that three non-associated individuals, tagged on different days, all started their migration on the same day raises the possibility of a coordinated timing of migration, at least to some degree. These observations need further investigation, but provide an interesting starting point for future studies.

Before starting to migrate northwards, all fin whales demonstrated a high level of site fidelity, indicating residency at the feeding ground. This fact, together with high densities and large aggregations observed at the feeding ground [13], underlines the special importance of the north coast waters of Elephant Island as a feeding habitat for fin whales [13]. The ‘Western Antarctic Peninsula and Islands’ have been recently declared as an Important Marine Mammal Area (IMMA) by the IUCN Marine Mammal Protected Areas Task Force possibly helping the implementation of appropriate protection measures [44,45].

## Data Availability

Data available from the Dryad Digital Repository: http://dx.doi.org/10.5061/dryad.4qrfj6qcg [46]. Additional data are provided in electronic supplementary material [47].
